# Estimation of Genetic Parameters of Heat Tolerance for Production Traits in Canadian Holsteins Cattle

**DOI:** 10.3390/ani12243585

**Published:** 2022-12-19

**Authors:** Ivan L. Campos, Tatiane C. S. Chud, Gerson A. Oliveira Junior, Christine F. Baes, Ángela Cánovas, Flavio S. Schenkel

**Affiliations:** 1 Centre for Genetic Improvement of Livestock, Department of Animal Biosciences, University of Guelph, Guelph, ON N1G 2W1, Canada; 2 Institute of Genetics, Vetsuisse Faculty, University of Bern, 3012 Bern, Switzerland

**Keywords:** dairy cow, heat stress, production, temperature-humidity index

## Abstract

**Simple Summary:**

Heat stress is a major problem in dairy cattle and has several negative consequences for both health and production. The possibility to genetically select for more heat tolerant animals can provide additional means for the dairy industry to address this environmental issue. When production records and environmental information, such as temperature and relative humidity are available, the genetic merit of the animals for milk production can be estimated under different environmental conditions. Animals can then be ranked according to their genetic merit under heat stress conditions for selection purpose. Therefore, the objective of this study was to estimate the genetic parameters for heat tolerance of milk, fat, and protein yields in Canadian Holstein cows. The correlation between the estimated genetic merit under thermal comfort and heat stress conditions indicated an antagonistic relationship between the level of production and heat tolerance. However, this correlation was moderate, which suggests that there exist animals with a high genetic merit for production that are also heat tolerant, which allows for genetic selection for heat tolerance to be carried out while still improving production. The results of this study support genetic selection for heat tolerance contributing towards the goal of improving the overall heat tolerance of Canadian Holstein cattle.

**Abstract:**

Understanding how cows respond to heat stress has helped to provide effective herd management practices to tackle this environmental challenge. The possibility of selecting animals that are genetically more heat tolerant may provide additional means to maintain or even improve the productivity of the Canadian dairy industry, which is facing a shifting environment due to climate changes. The objective of this study was to estimate the genetic parameters for heat tolerance of milk, fat, and protein yields in Canadian Holstein cows. A total of 1.3 million test-day records from 195,448 first-parity cows were available. A repeatability test-day model fitting a reaction norm on the temperature-humidity index (THI) was used to estimate the genetic parameters. The estimated genetic correlations between additive genetic effect for production and for heat tolerance ranged from −0.13 to −0.21, indicating an antagonistic relationship between the level of production and heat tolerance. Heritability increased marginally as THI increased above its threshold for milk yield (0.20 to 0.23) and protein yield (0.14 to 0.16) and remained constant for fat yield (0.17). A Spearman rank correlation between the estimated breeding values under thermal comfort and under heat stress showed a potential genotype by environmental interaction. The existence of a genetic variability for heat tolerance allows for the selection of more heat tolerant cows.

## 1. Introduction

Many improvements in the production efficiency of dairy cattle were achieved as a result of genetic selection over the last decades. Advances in data recording technologies and analytical techniques have enabled the dairy industry to keep pace with changes in consumer demands and production systems by performing genetic evaluations for several traits [1,2]. For instance, genetic selection has contributed to the efficiency of the Canadian dairy industry as noted by an increase of about 20% in milk production when compared to a 2% increase in the number of cows over the past 10 years [3,4]. A selection for a higher productivity; however, has negatively impacted the animal’s heat environmental sensitivity [5,6,7], which has become an important concern due to global warming [8]. In high-producing cows, more metabolic energy is directed to production, resulting in a reduced ability to cope with a heat stress environment [9]. When environmental conditions hamper the ability of a cow to dissipate heat and maintain its thermal balance, the cow suffers from heat stress, which can lead to reduced health and performance, resulting in economic losses and reduced animal welfare [10,11,12].

Heat stress can be assessed by the temperature-humidity index (THI), which accounts for the combined effect of ambient temperature and relative humidity on the heat exchange mechanisms of mammals [13,14]. Data from the closest weather station to the farms can be used as a proxy for on-farm environmental conditions to calculate the THI. This index can then be associated with production records, such as test-day (TD) records, to investigate the relationship between cow performance and heat stress using data from several farms over several years [15,16,17]. In Canada, studies on dairy cattle have focused on understanding the impact of heat stress on milk production and have shown a considerable negative impact on the Canadian dairy industry [18,19,20], highlighting the need to implement measures to mitigate heat stress.

Among the strategies to mitigate heat stress, genetic selection could help identify more heat tolerant animals. Studies on dairy cattle have estimated genetic parameters for milk production traits under heat stress conditions and have shown that genetic variability exists among animals, which supports the possibility of selecting animals better adapted to hot environmental conditions [21,22].

Estimating genetic parameters for heat tolerance requires a heat-stress indicator that should take into account the possible environmentally-induced variation in genotype expression (i.e., phenotypes), which is referred to as genotype by environment (GxE) interaction [23]. Therefore, studies have used reaction norms (RN) to model the expression of genotypes over an environmental descriptor (e.g., THI) on a continuous scale, as a unique curve [24]. This approach can be applied using random regression models (RRM) and includes a regression of the additive genetic effect on the environmental descriptor [25], thus estimating a genetic component for heat tolerance. Moreover, many studies have assumed an onset of heat stress based on a critical THI level, above which milk production starts to decline. This approach assumes a genetic effect within the thermal comfort zone and a genetic effect when the THI threshold is exceeded, describing a linear production response under heat stress conditions [26].

To the best of our knowledge, the genetic parameters of production traits considering heat stress have not yet been estimated in Canadian dairy cattle. In an initial study using Canadian Holstein dairy cows from two major dairy producing provinces (i.e., Quebec and Ontario) THI thresholds for milk production traits were determined [20]. Using the pre-determined thresholds, the objectives of this study were to estimate the genetic parameters for heat tolerance for milk, fat, and protein yields in Canadian Holsteins and to assess the potential presence of GxE interaction when accounting for heat tolerance in the genetic evaluation model.

## 2. Materials and Methods

### 2.1. Ethics and Data Availability

Ethics approval was not required by the institutional research ethics board of the University of Guelph for this study. Weather station data are publicly available and were retrieved from the Environment and Climate Change Canada’s online database (www.ec.gc.ca, accessed on 17 December 2022). The production data used in this study were made available by Lactanet (Guelph, ON, Canada; www.lactanet.ca, accessed on 17 December 2022). The data are recorded by producers and producer organizations following the requirements for dairy farmers as provided in the Code of Practice for the care and handling of farm animals [25].

### 2.2. Milk Production and Weather Data

A total of 1.3 million test-day (TD) records for milk, fat, and protein yields from 195,448 first-parity Holstein cows raised in Ontario and Quebec were provided by Lactanet. These two provinces account for about 70% of the milk production in Canada [3], with an average of 86 cows per farm and milk production of 11,053 kg per lactation [3]. Aiming to look at selection for heat tolerance on a national scale, the two provinces were analyzed together using average thresholds for the onset of heat stress across the two provinces. The data collected spanned a 10-year period from January 2010 to December 2019. Cows were required to have a minimum of four TD records for each trait collected within the interval from 5 to 305 days in milk (DIM). Only cows with ages at calving between 18 to 40 months were kept. Herds were required to have records from a minimum of five cows per year for at least five years. Pedigree information was available for cows with production records and included 952,076 animals.

Meteorological data from public weather stations registered in the Environment and Climate Change Canada (ECCC) website were obtained using the “weathercan” [27] package in R [28]. Hourly measurements (i.e., 24 records per day) of ambient temperature (AT, °C) and relative humidity (RH, %) were available. Weather stations were required to have a minimum of one reading per period (i.e., morning (5 a.m.–11 p.m.), afternoon (12 p.m.–8 p.m.), and night (9 p.m.–4 a.m.)) each day over the entire 10-year period. The daily THI was calculated by using maximum AT (AT_max) and minimum RH (RH_min) following Ouellet et al. [29]:

THI = (1.8 × AT_max + 32) − [(0.55 − 0.0055 × RH_min) × (1.8 × AT_max − 26)].

Studies have shown that when using weather station data as a surrogate to on-farm environmental conditions, the THI calculated using maximum daily AT and minimum daily RH better describes the conditions inside the barn when compared to the THI calculated using average measurements [3,13].

**Figure 1:**
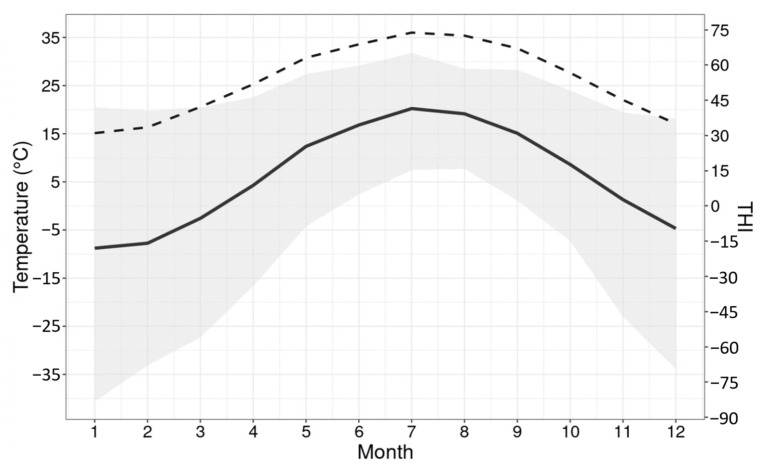
Distribution of mean ambient temperature (solid line) and its variation (shaded area) and mean temperature-humidity index (THI; dashed line) per month from January 2010 to December 2020.

The closest weather station was assigned to each farm based on their geographic coordinates using the “geosphere” [30] R package. Weather records were collected from a total of 55 weather stations located within a maximum distance of 20 km from each herd (average distance of 12.8 ± 4.9 km), with 49 weather stations linked to more than one herd. In this case, records from the closest weather station to each herd were used. To account for the delayed effect of heat load on production traits [31,32] the THI assigned for each TD (THI_TD) was the average THI on the day of milk recording and the two previous days (i.e., up to 48 h before milk recording) [33]. The average ambient temperature and THI over the months in the 10-year period are shown in Figure 1.

After merging production and weather records, the final data set contained 1.3 million TD records for the three production traits from 198,292 first-lactation cows in 1132 herds. The descriptive statistics for milk, fat and protein yields over the 10-year period below and above the THI threshold of each trait based on Campos et al. [20] are reported in Table 1. The adjusted means for all three traits for each THI value can be seen in the figures 3 to 5 in Campos et al. [20].

### 2.3. Statistical Analyses

An RN repeatability model was used to estimate the genetic parameters following Ravagnolo and Misztal. [19]. A linear function of THI above the THI threshold, i.e., the heat stress function was defined as the environmental descriptor to estimate the additive genetic effects of a cow under heat stress. The covariates of the heat stress function (HS) were defined as:

$$\mathrm{HS}=\left\{\begin{array}{c} \;0\;\;\;\;\;\;\;\;\;\;\;\;\;\;\;\;\;\;\;\;\;\;\;\;\;\;\;\;\;\mathrm{if}\;{\mathrm{THI}}_{\mathrm{TD}}\;\leq \;{\mathrm{THI}}_{\mathrm{threshold}}\\ {\mathrm{THI}}_{\mathrm{TD}}-{\mathrm{THI}}_{\mathrm{threshold}}\;\;\;\;\;\;\;\;\;\;\;\;\;\;\mathrm{if}\;{\mathrm{THI}}_{\mathrm{TD}}>{\mathrm{THI}}_{\mathrm{threshold}} \end{array}\right.$$

where THI_threshold_ value for milk, fat, and protein yields were 70, 57, and 58, respectively, based on Campos et al. [20], having just one single average threshold per trait across the two provinces. This function follows for the assumption that heat stress occurs only above a critical THI level and that the effect of heat stress is linear afterwards. The univariate animal random regression model was:

$$y_{\mathrm{ijklm}}=\mu +{\mathrm{AgeC}}_{i}+{\mathrm{DIM}}_{j}+{\gamma \mathrm{THI}}_{m}+a_{l}+\varphi_{l}{\left(\mathrm{HS}\right)}_{m}+{\mathrm{pe}}_{l}+\pi_{l}{\left(\mathrm{HS}\right)}_{m}+{\mathrm{HY}}_{k}+e_{\mathrm{ijklm}}$$

where $y_{\mathrm{ijklm}}$ is the TD record of milk, fat, or protein yields; μ is the overall mean; ${\mathrm{AgeC}}_{i}$ is the fixed effect of the *i*th age at calving grouped into every two months (11 classes); ${\mathrm{DIM}}_{j}$ is the fixed effect of the jth DIM grouped into every 30 days (11 classes); γ is the linear coefficient of the fixed linear regression of y on the environmental covariable (THI); THI_m_ is the THI_TD_ value of the *m*th TD record; $a_{l}$ and $\varphi_{l}$ are intercept and linear coefficient of the additive genetic random regression (i.e., the general additive genetic effect under thermal comfort and additive genetic effect for heat tolerance under heat stress, respectively); similarly, ${\mathrm{pe}}_{l}$ and $\pi_{l}$ are intercept, and the linear coefficient of the random regression for the permanent environmental effect of the *l*th cow, under thermal comfort and heat stress, respectively; ${\left(\mathrm{HS}\right)}_{m}$ is the heat stress function for the *m*th TD record; ${\mathrm{HY}}_{k}$ is the *k*th random effect of herd-year; and $e_{\mathrm{ijklm}}$ is the random residual term.

The random terms were assumed to be normally distributed with mean zero and a variance-covariance structure as follows:

$$Var=\left[\begin{array}{c} a\\ \varphi \\ pe\\ \pi \\ hy\\ e \end{array}\right]=\left[\begin{array}{cccccc} A\sigma_{a}^{2} & A\sigma_{a,\varphi } & 0 & 0 & 0 & 0\\ A\sigma_{a,\varphi } & A\sigma_{\varphi }^{2} & 0 & 0 & 0 & 0\\ 0 & 0 & I\sigma_{\mathrm{pe}}^{2} & I\sigma_{\mathrm{pe},\pi } & 0 & 0\\ 0 & 0 & I\sigma_{\mathrm{pe},\pi } & I\sigma_{\pi }^{2} & 0 & 0\\ 0 & 0 & 0 & 0 & I\sigma_{\mathrm{hy}}^{2} & 0\\ 0 & 0 & 0 & 0 & 0 & I\sigma_{e}^{2} \end{array}\right]$$

where **A** is the numerator relationship matrix; **I** is the identity matrix; $\sigma_{a}^{2}$ and $\sigma_{\varphi }^{2}$ are the variances for the general additive genetic effect and additive genetic effect of heat tolerance, respectively; $\sigma_{\mathrm{pe}}^{2}$ and $\sigma_{\pi }^{2}$ are the variances for the general permanent environmental effect and permanent environmental of heat tolerance, respectively; $\sigma_{a,\varphi }$ and $\sigma_{\mathrm{pe},\pi }$ are the covariances between general and heat tolerance of a and pe effects, respectively; $\sigma_{\mathrm{hy}}^{2}$ is herd-year variance; and $\sigma_{e}^{2}$ is the residual variance.

Variance components were estimated using average information REML implemented in AIREMLF90 [34]. Heritability was estimated as follows:

$$h^{2}=\frac{\sigma_{a}^{2}+2\left(\mathrm{HS}\right)\sigma_{a,\varphi }+{\left(\mathrm{HS}\right)}^{2}\sigma_{\varphi }^{2}}{\sigma_{a}^{2}+2\left(\mathrm{HS}\right)\sigma_{a,\varphi }+{\left(\mathrm{HS}\right)}^{2}\sigma_{\varphi }^{2}+\sigma_{\mathrm{pe}}^{2}+2\left(\mathrm{HS}\right)\sigma_{\mathrm{pe},\pi }+{\left(\mathrm{HS}\right)}^{2}\sigma_{\pi }^{2}+\sigma_{\mathrm{hy}}^{2}+\sigma_{e}^{2}}$$

Genetic correlation between additive genetic effect and additive genetic effect of heat tolerance was estimated as:

$$\mathrm{corr}\left(a,\left(\mathrm{HS}\right)\sigma_{\varphi }^{2}\right)=\frac{\left(\mathrm{HS}\right)\sigma_{a,\varphi }}{\sqrt{\sigma_{a}^{2}\times {\left(\mathrm{HS}\right)}^{2}\sigma_{\varphi }^{2}}}$$

### 2.4. Estimated Breeding Value and GxE Interaction

With variance components estimated, the estimated breeding value (EBV) of each animal was computed and EBV under heat stress was obtained as EBV*_l_* = ${\hat{a}}_{l}$ + (HS) × ${\hat{\varphi }}_{l}$, where ${\hat{a}}_{l}$ and ${\hat{\varphi }}_{l}$ are the predicted solutions for the general additive effect and additive effect of heat tolerance (i.e., heat stress conditions) of the *l*th animal, respectively. To investigate the presence of GxE interaction, a Spearman rank correlation was estimated to compare the rank of bull’s EBV that had a reliability ≥0.70 (following the criteria for official publication of national evaluations, [35]) under thermal comfort and under heat stress (5 and 10 units above the THI threshold of each trait). In addition, rank correlation was estimated between the top 100 bull’s EBV under thermal comfort and under heat stress. Finally, to illustrate the presence of a GxE interaction and the re-ranking of bulls, the RN of the top 10 bulls based on their predicted EBV for milk components were plotted against the THI gradient.

## 3. Results

### 3.1. Milk Production and Weather Conditions

**Figure 2:**
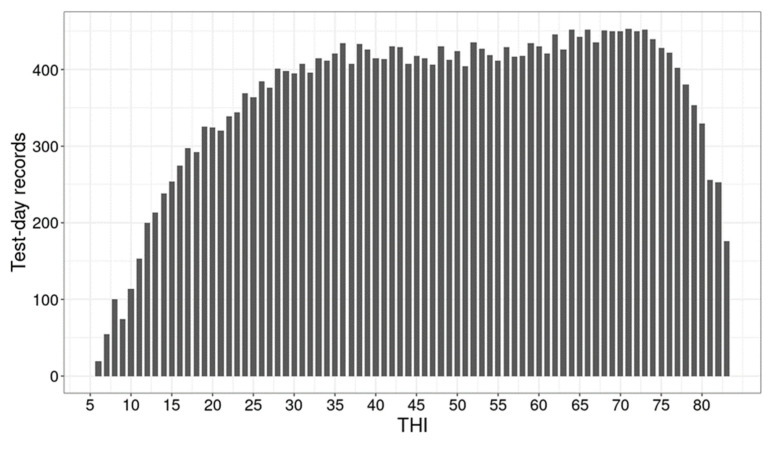
Number of test-day records per temperature-humidity index (THI).

The mean and variation in ambient temperature and the mean THI are presented in Figure 1. A noticeable decline in overall milk production traits under heat stress were observed (Table 1), particularly for fat and protein yields, indicating an impact of heat stress on a cow’s performance. Considering an average THI threshold for milk production traits (i.e., THI 62), approximately 40% of TD records were obtained under heat stress conditions (THI > 62). A total of 190,039 cows had TD records under both thermal comfort and heat stress conditions. The median of repeated TD records per cow under and above the average THI threshold were four and three, respectively. The number of TD records per THI value is shown in Figure 2.

### 3.2. Variance Component Estimates

Variance component and heritability estimates for milk, fat, and protein yields are presented in Table 2. Variance components can be estimated along the THI gradient; thus, the maximum THI (THI 83) observed in our data allows for the observation of the behavior of each component under severe heat stress conditions. Estimated genetic correlations between the additive genetic effect under thermal comfort and the additive genetic effect for heat tolerance were all low to moderate and negative (−0.13 to −0.21). Heritability estimates increased marginally as THI increased above the THI threshold for milk yield (0.21 to 0.23) and protein yield (0.14 to 0.16), remaining constant for fat yield (0.17).

### 3.3. Estimated Breeding Value and GxE

Descriptive statistics of all bulls’ (n = 17,498) estimated breeding values for milk, fat, and protein yield are presented in Table 3. The overall mean of the estimated additive effects of heat tolerance (linear regression coefficients) was negative for all traits, indicating the negative impact of heat stress on the genetic potential for milk production. The Spearman rank correlation between all bulls’ EBV with a reliability ≥0.70 (n = 1916) under thermal comfort and under heat stress conditions is presented in Table 4. The correlation obtained when THI was five and ten units above the threshold of each trait was >0.95, indicating an overall minimum re-ranking of bulls. However, when only the top 100 bull’s EBV were considered, rank correlation was as low as 0.59, 0.82, and 0.72 for milk, fat, and protein yields, respectively.

**Figure 3:**
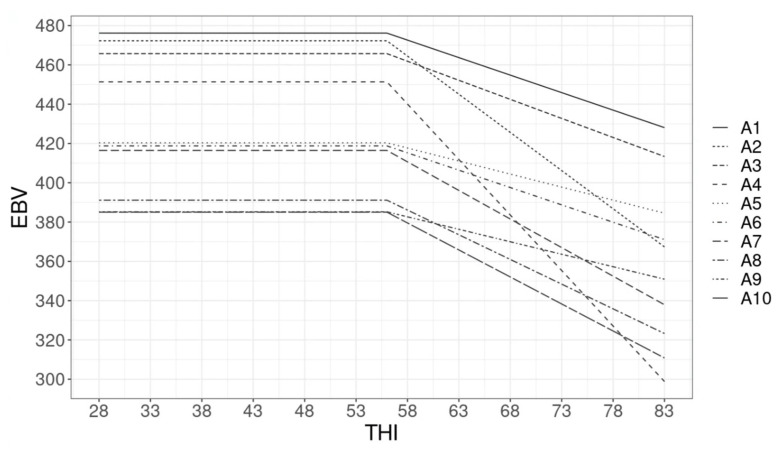
Top 10 bulls’ estimated breeding value (EBV) for fat yield as a function of the temperature-humidity index (THI).

**Figure 4:**
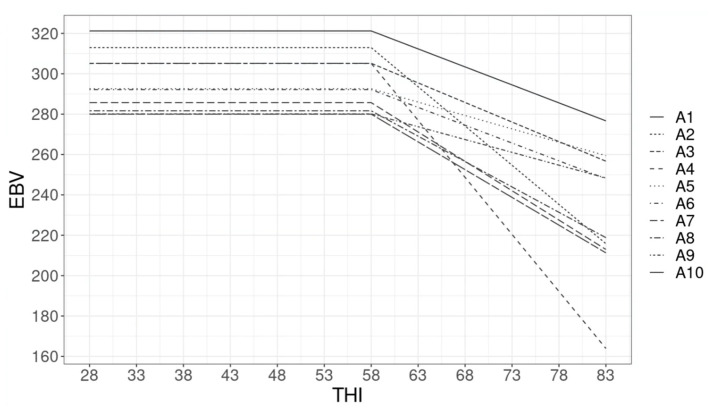
Top 10 bulls’ estimated breeding value (EBV) for protein yield as a function of the temperature-humidity index (THI).

The EBVs for protein and fat yields of the top 10 bulls (ranked based on the predicted EBV for production under thermal comfort) are presented in Figure 3 and Figure 4. Re-ranking of bulls based on their EBV for fat and protein yields was observed. For instance, for protein yield, the bulls that rank second (B2) and fourth (B4) under thermal comfort rank sixth and tenth at THI 70. The steep decline in EBV after the THI threshold depicts bulls with daughters that are genetically less heat tolerant.

## 4. Discussion

### 4.1. Milk Production and Weather Conditions

The present study aimed to estimate the genetic parameters for heat tolerance for milk production traits in Canadian Holsteins cattle. Test-day records of milk, fat, and protein yields were merged with THI measurements obtained from the closest weather station to each farm. The provinces where the farms were located (Ontario and Quebec) comprise the Central Canada region and are predominantly characterized by high humidity and large seasonal variation in temperatures throughout the year. During the summer, cows are exposed to around 156 days of heat stress conditions (THI > 62) in Ontario and Quebec [20]. Many studies have highlighted the negative effects of high heat loads on production traits. In Canada, studies have shown that milk components are more affected by heat stress than milk production [19,20], which highlights the importance of addressing this environmental challenge, as Canadian producers are paid based on fat and protein yields [36].

### 4.2. Variance Component Estimates

Reaction norm modeling was used through RRM to estimate the genetic parameters for production traits considering heat stress. The model fitted the additive genetic and permanent environmental effects as a function of THI, considering that when a critical THI level is exceeded cows start being affected by heat stress. Thus, a general additive genetic variance and an additive genetic variance for heat tolerance were estimated. The latter variance depends on how many THI units above the heat stress threshold are considered.

The estimated genetic correlations between additive genetic effect for production and for heat tolerance were negative (unfavorable), which is in line with other studies. The correlations estimated in the present study are smaller than those found for first-parity Holstein cows in the USA (−0.25 to −0.46) [37,38] and Italy (−0.31 to −0.51) [17], but similar to those observed in Belgium (−0.16 to −0.18) [39]. The antagonistic relationship between overall production levels and heat tolerance shows that, particularly for fat and protein yield, selecting for higher production is detrimental to heat tolerance, and this may be aggravated in regions with an overall high temperature and humidity throughout the year.

Heritability estimates under thermal comfort were in line with estimates obtained in other studies using a similar RN model [17,39,40]. Some studies have associated changes in heritability estimates with differences in expected genetic gain over the THI values [41,42,43]. For instance, Santana et al. [41] observed a decrease in heritability estimates for milk yield as THI increased, indicating that a higher response to selection may be expected under thermal comfort. The differences found in our study are most likely related to the nature of the random regression model [21]. Considering their magnitude, they do not indicate changes in response to selection for these traits depending on temperature and humidity conditions. Nonetheless, factors specific to the studies, such as population, herd locations, and models used, may result in a different behavior of genetic parameter estimates across THI levels.

### 4.3. Estimated Breeding Value and GxE

The negative impact of heat stress on the genetic potential for milk production was observed in the overall negative mean of the estimated additive effects of heat tolerance for each trait (Table 3). However, the presence of some positive additive effects for heat tolerance implies the possibility of selecting animals genetically more heat tolerant. Thus, genetic selection provides an additional means to maintain or even improve the productivity of the dairy industry facing a changing environment.

The existence of GxE interaction may result in the re-ranking of animals based on their estimated genetic merit in different environments. Spearman rank correlations between EBVs under thermal comfort and five or ten units above the threshold were generally high, except when only the top 100 bulls were considered (Table 4). Considering the low number of elite sires selected for breeding, re-ranking is expected under different environmental conditions (thermal comfort vs. heat stress) in the studied population. Studies investigating GxE interactions, resulting in the re-ranking of animals’ EBV under different environmental conditions vary in the literature. For instance, Carrara et al. [43], observed a Spearman rank correlation of less than 0.70 between EBV at different THI levels for several milk traits, such as milk yield and casein percentages. Bernabucci et al. [17] also reported changes in the ranking of sires when including THI in genetic evaluations. Conversely, studies investigating heat stress based on THI [39] or regional climate profiles [33] reported genetic correlations >0.80, suggesting low re-ranking of sires in different environmental conditions.

The presence of a GxE interaction was also observed by the inspection of the predicted EBVs across the THI gradient. Given the antagonistic relationship between heat tolerance and production, animals with superior genetics for production under thermal comfort zones may have a low genetic merit for heat tolerance and, therefore, may rank lower at high THI levels. For fat yield (Figure 4), less re-ranking among the top 10 bulls was observed when compared to protein yield, which is in line with the higher rank correlation between EBVs for fat yield at different THI levels. Particularly for this trait, GxE interaction does not seem to play an important role when considering different levels of THI. Most likely the genes involved in fat yield under thermal comfort conditions are the same under heat stress conditions.

In the present study, no information was available on the implementation of on-farm heat abatement measures, and climate variables were collected from the closest weather station to each farm, which may have led to a potential underestimation of the actual heat stress on the farms [29,44]. However, considering the limited access to on-farm weather data, weather stations were a useful source of information, particularly for genetic studies into heat tolerance [45]. Another important aspect of this study is the use of TD records. When using TD records, the cumulative effect of heat stress cannot be captured; thus, it is potentially underestimated [33,45]. However, when more frequent records (e.g., daily records) are not widely available, an adequate estimation of the overall effect of heat stress can still be obtained with a large number of TD records per THI value.

The model used in the present study assumed a population THI threshold and, therefore, that the onset of heat stress occurs at the same THI value for all animals. Since there is variability in how animals cope with heat, and some animals are more heat tolerant than others, they may show a different onset of heat stress as well. However, models allowing for different thresholds for each animal (or using higher order ordinary or orthogonal polynomials in random regression models) are computationally demanding and may not be practical for a national genetic evaluation for heat tolerance. In addition, a possible interaction between THI and different stages of lactation (i.e., DIM) may exist [42,46], which was not accounted for in the current study. The repeatability model used in this study also assumed that the breeding values for production traits do not vary with DIM, which is consistent with the assumption of a genetic correlation of one between records in the repeatability model.

This is a pioneering study investigating the possibility to improve the overall heat tolerance of dairy cattle in Canada by identifying animals genetically more heat tolerant. Moving forward, a sequel study, including other provinces across Canada should be conducted. Heat tolerance of multiparous cows should also be investigated since they are more sensitive to heat stress than primiparous cows [17,20,37]. In addition, the effect of heat stress on economically important traits, other than production traits, should be investigated.

## 5. Conclusions

The reported negative genetic correlation between the overall additive effect and the additive genetic effect for heat tolerance is in line with other studies conducted in other countries. Therefore, the continued genetic selection for production will result in a greater susceptibility to heat stress. However, this negative genetic correlation is only moderate, which, associated with the existing genetic variability for heat tolerance, would allow for selection for more heat tolerant cows, while maintaining or even improving production. Our study also showed a potential re-ranking of top bulls particularly for milk and protein yields at high THI values, which indicates some level of genotype by environment interaction. This study is part of the initial research efforts investigating the possibility of genetically selecting heat tolerant Holstein cows in Canada.

## Figures and Tables

**Table 1:** Mean and standard deviation (SD) of test-day records for milk production traits below and above the temperature-humidity index (THI) threshold for milk, fat and protein yields.

Trait	THI ≤ THI_threshold_^1^		THI > THI_threshold_^1^	
	Mean	SD	Mean	SD
Milk yield (kg/d)	30.26	6.27	30.12	6.37
Fat yield (g/d)	1190.88	254.02	1147.90	252.78
Protein yield (g/d)	987.42	188.88	963.90	190.19

^1^ Milk yield THI threshold: 70; Fat yield THI threshold: 57; Protein yield THI threshold: 58.

**Table 2:** Estimates of variance components and heritability ($h^{2}$ ) for milk, fat, and protein yields below temperature-humidity index (THI) threshold and at maximum THI, and genetic correlation ($r_{g}$ ) between general additive genetic effect and additive genetic effect for heat tolerance.

	Milk Yield (kg^2^)		Fat Yield (g^2^)		Protein Yield (g^2^)	
Parameter ^1^	THI < 70	THI = 83	THI < 57	THI = 83	THI < 58	THI = 83
$\sigma_{a}^{2}$	7.30 (±0.12)	7.30	10,675.00 (±203.69)	10,675.00	4874.80 (±106.31)	4874.80
$\sigma_{\varphi }^{2}$	0.02 (±0.01)	3.95	5.89 (±0.50)	4297.60	5.08 (±0.34)	3439.62
$\sigma_{\mathrm{pe}}^{2}$	6.98 (±0.08)	6.98	10,660.00 (±141.23)	10,660.00	6872.50 (±76.07)	6872.50
$\sigma_{\pi }^{2}$	0.03 (± 0.01)	7.21	24.55 (±0.51)	17,902.05	17.74 (±0.32)	11,998.32
$h^{2}$	0.21 (± 0.01)	0.23	0.17 (± 0.01)	0.17	0.14 (± 0.01)	0.16
$r_{g}$	−0.13 (± 0.01)		−0.21 (± 0.01)		−0.20 (± 0.01)	

^1^${\;\sigma }_{a}^{2}$: general additive genetic variance; $\sigma_{\varphi }^{2}$: additive genetic variance for heat tolerance; $\sigma_{\mathrm{pe}}^{2}$: general permanent environmental variance; $\sigma_{\pi }^{2}$: permanent environmental variance for heat tolerance.

**Table 3:** Estimated breeding values under thermal comfort (EBV_TC), heat tolerance (EBV_HT), and at five (EBV_HS5) and ten (EBV_HS10) units above the temperature-humidity index threshold (i.e., EBV_TC + 5 × or 10 × EBV_HT, respectively) for milk, fat, and protein yields.

	Milk Yield (kg)			Fat Yield (g)			Protein Yield (g)		
Item	Min	Mean	Max	Min	Mean	Max	Min	Mean	Max
EBV_TC	−7.09	1.33	10.82	−146.50	64.63	476.17	−147.28	50.43	325.06
EBV_HT	−0.24	−0.02	0.22	−8.23	−2.14	1.93	−5.64	−1.34	4.10
EBV_HS5	−6.94	1.24	10.13	−155,88	53.93	453.04	−162.41	43.69	312.30
EBV_HS10	−6.80	1.15	10.92	−170.18	43.23	429.92	−177.54	36.94	303.40

**Table 4:** Spearman rank correlation between estimated breeding value under thermal comfort (EBV_TC) and breeding values estimated on five units (EBV_HS5 = EBV_TC + 5 × EBV_HT) and ten units (EBV_HS10 = EBV_TC + 10 × EBV_HT) above the temperature-humidity index (THI) threshold of each trait.

	All Bulls ^1^ (n = 1916)		Top 100 Bulls	
Trait	EBV_TC/EBV_HS5	EBV_TC/EBV_HS10	EBV_TC/EBV_HS5	EBV_TC/EBV_THS10
Milk	0.98	0.95	0.85	0.59
Fat	0.99	0.99	0.96	0.82
Protein	0.99	0.98	0.91	0.72

^1^ Bulls with reliability ≥0.70.

## Data Availability

The data used in this study were provided by Lactanet (Guelph, ON, Canada; www.lactanet.ca, accessed on 17 December 2022) and cannot be shared. All the data supporting the results of this study are included in the article.
